# Seroprevalence of Brucellosis among Children in the Middle Anatolia Region of Turkey

**Published:** 2014-12

**Authors:** Serdar Gül, Özgün Kiriş Satilmiş, Baris Ozturk, Mehmet İlker Gökçe, Ferit Kuscu

**Affiliations:** ^1^Department of Infectious Diseases and Clinical Microbiology, Kirikkale University, Kirikkale, Turkey; ^2^Department of Microbiology and Clinical Microbiology, Sorgun Hospital, Yozgat, Turkey; ^3^Department of Infectious Diseases and Clinical Microbiology, Ulucanlar Eye Education and Research Hospital, Ankara, Turkey; ^4^Department of Urology, Sorgun Hospital, Yozgat, Turkey; ^5^Department of Infectious Diseases and Clinical Microbiology, Numune Education and Research Hospital, Adana, Turkey

**Keywords:** Brucellosis, Seroprevalence, Turkey

## Abstract

Brucellosis is an important public-health problem in Turkey. Children may constitute 20 to 30% of all brucellosis cases in the world, especially in the endemic regions. Data on the seroprevalence of brucellosis in childhood are very limited. In this study, we aimed to evaluate the seroprevalence of brucellosis among a child population. One thousand one hundred and ten subjects were included in the study. Blood samples were collected and tested with Rose Bengal (RB) and standard tube agglutination test (SAT). RB test results were positive for 6 patients, and SAT was negative for all patients. Our findings suggest that seroprevalence of brucellosis is decreasing in Middle Anatolia due to a new cattle vaccination and eradication programme which was initiated in 2009.

## INTRODUCTION

*Brucella* species are small, Gram-negative coccobacilli, which are non-motile, non-spore-forming and encapsulated (1). *Brucella* are zoonotic pathogens typically associated with abortion in sheep, goats, pigs, and cattle. *Brucella melitensis*, *Brucella abortus*, and *Brucella suis* are usually responsible for human disease (2). Brucellosis, a debilitating febrile illness, can develop in humans following direct contact with infected animals or consumption of contaminated milk and milk products. The disease can rarely spread from human to human. The disease can cause serious morbidity, and it has important economic consequences (3). Diagnosis of brucellosis is based on clinical and laboratory findings. As the signs and symptoms are not pathognomonic for brucellosis, serological and bacteriological tests are essential to confirm the diagnosis.

Brucellosis is an important public-health problem in many developing countries, such as Turkey, especially in the Middle Anatolia region (2). The seroprevalence of brucellosis varies between 2% and 6% in Turkey (4). In some high-risk groups, this rate may increase up to 12.5% (5,6). Children may constitute 20 to 30% of all brucellosis cases in the world, especially in the endemic regions (1). Data on the seroprevalence of brucellosis in child population are very limited. The objective of this study was to research brucellosis seroprevalence in 7 districts of Yozgat province in Turkey. Yozgat province is located in Middle Anatolia and has 14 districts, including Sorgun. Sorgun State Hospital is the reference hospital for the 7 districts included in the study. Sorgun State Hospital serves about 250,000 people, approximately 90,000 of them being aged below 18 years. Public health laboratory records of Yozgat province indicate that 778 people were diagnosed with brucellosis between 2007 and 2013 in Yozgat province, and 107 of them were below 18 years of age. An effective vaccine for human beings is not yet available; thus, protection efforts through pasteurization of dairy products and brucellosis control and eradication programmes in animals are essential.

## MATERIALS AND METHODS

This study was conducted in 7 districts of Yozgat province between June and September 2012. Yozgat province is located in the Middle Anatolia region where brucellosis is endemic. We aimed to examine the prevalence of brucellosis among children in this area.

A simple random-sampling method was used. Patients were selected among children aged less than 18 years in the 7 districts of Yozgat, who were admitted to Sorgun State Hospital due to non-infectious health problems. Children with fever, leukocytosis/leukopenia or those with possibility of infection were not included in the study to prevent selection bias. Children who had an immune-suppressing disease, were receiving immune-suppressing medication, or who refused to participate in the study were also excluded. Data were collected on age, sex, and family history of brucellosis. Serum samples were obtained from centrifugation of peripheral blood and preserved at −40 ^0^C until the day of examination. All the serum samples were studied with Rose Bengal slide agglutination test (RB) and standard tube agglutination test (SAT). All sera were routinely diluted from 1/20 to 1/1,280. Each batch of the test included a positive control and a negative saline control. A definite agglutination of the suspension was considered a positive reaction. Agglutination was not seen in the negative samples. For the positive samples, the lowest positive titre was determined. Titrations of 1/80 and over were accepted as positive for SAT (3,7).

The study was approved by the local Ethical Board of Bozok University Faculty of Medicine. The subjects and their parents were informed about the nature of the study, and written consents were obtained from the parents of patients.

Statistical analyses were done using the SPSS software (version 15.0) (SPSS Inc., Chicago, IL, USA). The mean age was presented as mean±standard deviation (M±SD).

## RESULTS

Data from 1,110 children were analyzed. Mean age of the study population was 8.4±6.4 years (range 0-18). There were 591 females (53.2%) and 519 males (46.8%). These 1,110 children belonged to 1,110 families meaning that there was no sister or brother. When the families of these children were screened, 335 (30.2%) families were occupied in animal breeding. Additionally, 245 (73.2%) of these 335 families were found to produce their own cheese and, of these families, only 86 (35.1%) reported to have boiled milk before making cheese while rest of them only warmed up the milk to a certain degree sufficient for cheese production. Abortions in animals were reported in 75 (22.3%) of the families occupied in the field of animal breeding.

In the serological examinations, RB test results were positive for 6 patients, and SAT results were negative for all patients.

## DISCUSSION

Brucellosis is endemic in Western Asia, Middle East, Africa, Latin America and Mediterranean regions. Turkey is located in both Mediterranean and Western Asia regions, and brucellosis has been an important public-health problem. The first documented case of laboratory-confirmed brucellosis in Turkey was reported in 1915 (8). *B. melitensis* accounts for the majority of the brucellosis cases in Turkey, followed by *B. abortus*. *B. suis* is not frequently seen because pig farming is uncommon due to religious reasons in Turkey (2).

The seropositivity of brucellosis was found to be 3.2% in the elderly population (over 65 years of age) in the Middle Anatolia region of Turkey (3). The high prevalence in the Middle Anatolia region is associated with the frequent consumption of fresh cheese and butter produced from unpasteurized milk.

Brucellosis is not uncommon in children, and people of all ages are susceptible to the disease. In a study that examined the symptomatic active brucellosis cases in Van, Eastern Anatolia, 3.6% of 1,028 patients were between the age of 3 and 12 years (9). In a retrospective review of 90 paediatric cases in Ankara, the capital city, 52 patients (57.8%) were from rural areas and 38 (42.2%) from urban areas (10). Consumption of fresh cheese was the mode of transmission in 64 children (71.1%). Parents of 41 children (45.6%) worked in farms. There was a positive family history of brucellosis in 14 (15.6%) children (10). Similarly, in another retrospective study conducted on children of 20 months to 16 years of age diagnosed with brucellosis, positive family history was reported in 13.5% of the cases (11).

Occupational exposure is an important risk factor. For instance, seropositivity in butchers is 2-12.5%, with 0.8-11.9% in cattle dealers and slaughter-house workers (5-6). The seropositivity among farmers is 6.2% in Turkey (12). Seroprevalence in rural areas and dairy farms were found to be 7.2 and 5.2% respectively (13,14). In another study, the prevalence is reported to be 4.8% in rural areas of Western Anatolia (15).

Children older than 5 years constitute the majority of childhood brucellosis cases. This is associated with a higher probability of working for animal care and consumption of unpasteurized milk (10).

Because of the social difficulties and geographical structure of the region in our study, the serum samples were obtained from patients at the hospital rather than homes. This procedure may have caused selection bias in favour of high prevalence of brucellosis. This is the main limitation of our study, and the results should be considered in light of this possible bias. However, none of the subjects was diagnosed with brucellosis. The prevalence of brucellosis decreased in Turkey as a result of animal disease control programmes directed by the Ministry of Agriculture and Rural Affairs. Brucellosis eradication programmes were started in the early 1950s as a ‘test and slaughter’ programme. This programme was unsuccessful. A more successful vaccination campaign was started in the 1960s, followed by other programmes in the 1970s and 2000s. With the help of these vaccination campaigns, seropositivity decreased for both human and animal brucellosis (2). Recently, following directives of the European Community Council on animal health problems affecting intra-community trade in bovine and ovine animals, a new national brucellosis control and eradication project was initiated in Turkey on 3 April 2009 (2).

In our study, none of the patients in the childhood period was positive for SAT while the RB test had provided 6 positive results. RB test generally is used as a screening method due to its low specificity. Therefore, positive RB test results should be confirmed with the SAT having the higher specificity.

### Conclusions

According to the Public Health Laboratory Reports of Yozgat province, the number of new cases of brucellosis was 48, 27, and 7 in the Yozgat province in 2010, 2011, and 2012 (January–June) respectively. On the other hand, the number of new brucellosis patients was 387, 209, and 98 in 2007, 2008, and 2009 in the Yozgat province respectively. The number of brucellosis cases with ages less than 18 years in Yozgat province were 64, 26, 10, 1, 4, and 2 in 2007, 2008, 2009, 2010, 2011, and 2012 respectively. These numbers indicate a sharp decrease in the number of new brucellosis patients since 2009. We should also note that there was already a decreasing trend before 2009 as well. While it can be due to the better hygiene and healthcare conditions, continuation of this trend in a stronger pattern after 2009 may be the result of the new eradication programme.
